# Vital Signs Directed Therapy: Improving Care in an Intensive Care Unit in a Low-Income Country

**DOI:** 10.1371/journal.pone.0144801

**Published:** 2015-12-22

**Authors:** Tim Baker, Carl Otto Schell, Edwin Lugazia, Jonas Blixt, Moses Mulungu, Markus Castegren, Jaran Eriksen, David Konrad

**Affiliations:** 1 Department of Anaesthesia, Intensive Care and Surgical Services, Karolinska University Hospital, Stockholm, Sweden; 2 Global Health, Health Systems & Policy, Department of Public Health Sciences, Karolinska Institutet, Stockholm, Sweden; 3 Department of Physiology and Pharmacology, Karolinska Institutet, Stockholm, Sweden; 4 Centre for Clinical Research Sörmland, Uppsala University, Uppsala, Sweden; 5 Department of Internal Medicine, Nyköping Hospital, Sörmland County Council, Nyköping, Sweden; 6 Department of Anaesthesia and Intensive Care, Muhimbili University of Health and Allied Sciences, Dar es Salaam, Tanzania; 7 Department of Anaesthesia and Intensive Care, Muhimbili National Hospital, Dar es Salaam, Tanzania; 8 Department of Laboratory Medicine, Division of Clinical Pharmacology, Karolinska Institutet at Karolinska University Hospital Huddinge, Stockholm, Sweden; Azienda Ospedaliero-Universitaria Careggi, ITALY

## Abstract

**Background:**

Global Critical Care is attracting increasing attention. At several million deaths per year, the worldwide burden of critical illness is greater than generally appreciated. Low income countries (LICs) have a disproportionally greater share of critical illness, and yet critical care facilities are scarce in such settings. Routines utilizing abnormal vital signs to identify critical illness and trigger medical interventions have become common in high-income countries but have not been investigated in LICs. The aim of the study was to assess whether the introduction of a vital signs directed therapy protocol improved acute care and reduced mortality in an Intensive Care Unit (ICU) in Tanzania.

**Methods and Findings:**

Prospective, before-and-after interventional study in the ICU of a university hospital in Tanzania. A context-appropriate protocol that defined danger levels of severely abnormal vital signs and stipulated acute treatment responses was implemented in a four week period using sensitisation, training, job aids, supervision and feedback. Acute treatment of danger signs at admission and during care in the ICU and in-hospital mortality were compared pre and post-implementation using regression models. Danger signs from 447 patients were included: 269 pre-implementation and 178 post-implementation. Acute treatment of danger signs was higher post-implementation (at admission: 72.9% vs 23.1%, p<0.001; in ICU: 16.6% vs 2.9%, p<0.001). A danger sign was five times more likely to be treated post-implementation (Prevalence Ratio (PR) 4.9 (2.9–8.3)). Intravenous fluids were given in response to 35.0% of hypotensive episodes post-implementation, as compared to 4.1% pre-implementation (PR 6.4 (2.5–16.2)). In patients admitted with hypotension, mortality was lower post-implementation (69.2% vs 92.3% p = 0.02) giving a numbers-needed-to-treat of 4.3. Overall in-hospital mortality rates were unchanged (49.4% vs 49.8%, p = 0.94).

**Conclusion:**

The introduction of a vital signs directed therapy protocol improved the acute treatment of abnormal vital signs in an ICU in a low-income country. Mortality rates were reduced for patients with hypotension at admission but not for all patients.

## Introduction

Critical Care is needed in all the countries in the world [1]. There is a huge, under-appreciated burden of critical illness exceeding the yearly number of deaths from diseases such as breast cancer, HIV/AIDS, and asthma [2]. The burden is expected to rise further with ageing and urbanised populations, the consequences of natural and man-made disasters and increasing surgical services [2–4]. Low and middle income countries have a disproportionately greater burden of critical illness with over 90% of global trauma deaths, maternal deaths and deaths from pneumonia, meningitis and other infections [5–7]. The recent Ebola outbreak in West Africa further emphasised the importance of critical care [8].

Critical care facilities are scarce in low-income countries (LICs). More than half of low-income countries lack published data on the number of Intensive Care Unit (ICU) beds. Nepal and Uganda report only 16.7 and 1.0 ICU beds per million inhabitants respectively [9, 10]. Hospitals in Africa do not have the resources to comply with international critical care guidelines for sepsis [11]. Tanzania, with a population of 49 million and an annual health expenditure of $42 per capita, is a LIC in East Africa [12]. Very few hospitals in Tanzania have ICUs, and there are deficiencies in infrastructure, human resources, training, and routines for critical care [13, 14]. Hospital care for sick patients is cost-effective even in the LIC setting [15–17] and the development of routines and interventions for critical care is needed for effective management of patients when resources are scarce [18, 19].

Abnormal vital signs (heart rate, blood pressure, respiratory rate, conscious level, oxygen saturation, body temperature) are antecedents to death and other adverse outcomes [20–22]. Much recent work in high-income countries has focused on Early Warning Scores [23], Rapid Response Teams [24] and Goal Directed Therapies [25, 26] that use vital signs to identify critical illness and trigger acute treatments. These initiatives have improved routines and reduced mortality [24, 27]. In Tanzania, admission Early Warning Scores and severely abnormal vital signs (danger signs) were associated with mortality [28, 29] and were rarely followed by acute treatments [30]. In this study we aimed to assess whether the introduction of a context-appropriate vital signs directed therapy protocol could improve the acute treatment of abnormal vital signs and reduce mortality for critically ill patients in Tanzania.

## Materials and Methods

We conducted a single-centre, prospective, before-and-after interventional study of vital signs, acute treatments and outcomes of patients in the Intensive Care Unit (ICU) at Muhimbili National Hospital (MNH) in Tanzania.

### Setting

MNH is a national referral hospital with 1500 beds, receiving patients from the Dar es Salaam area (4.5 million inhabitants) and hospitals nationwide. During the period of the study, MNH had 4 specialists in Anaesthesia & Intensive Care who were responsible for both the 6-bed ICU and the 16 operating theatres. There were 4–6 ICU nurses per shift in the ICU, none of whom had formal Intensive Care qualifications. The ICU had six ventilators, suction, piped oxygen and non-invasive monitoring. Crystalloids, antibiotics, blood transfusions, adrenaline and dopamine were used but noradrenaline, other inotropes and continuous sedation were not.

### Development of the protocol

**Fig 1:**
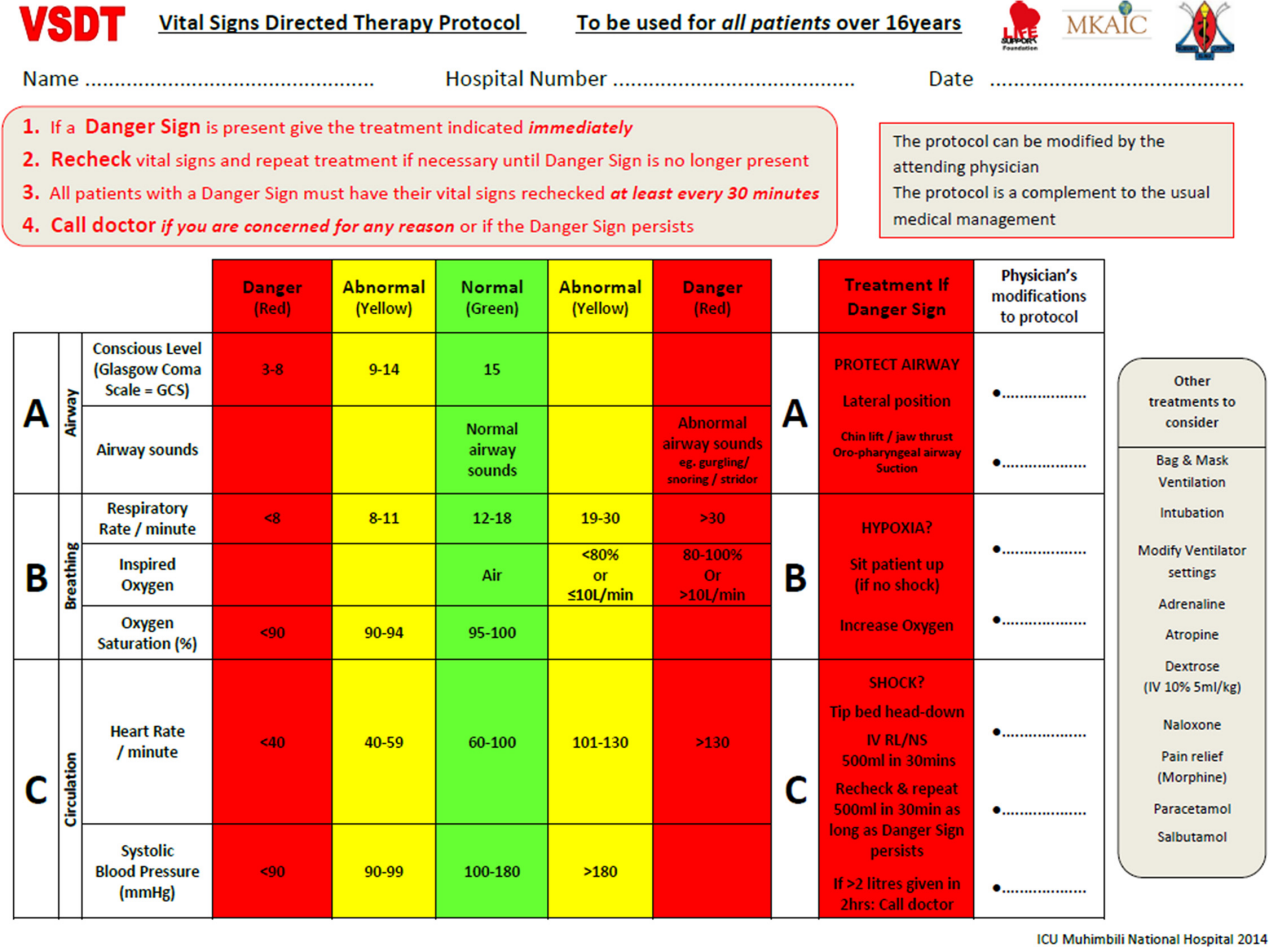
Vital Signs Directed Therapy Protocol.

The Vital Signs Directed Therapy (VSDT) protocol (Fig 1) was designed with the following requisites: feasible given health workers’ time constraints and resource availability; include actions that can be carried out by nurses without a physician’s immediate presence; modifiable by treating physicians; not be regarded as a replacement for clinical judgement; and include prompts for more advanced therapies. An expert group was formed, comprised of the researchers, six ICU nurses from Karolinska and Muhimbili and two experts in critical care in low-income countries from the European Society of Intensive Care Medicine (see acknowledgments). An iterative process was conducted with review and amendment of draft protocols. The researchers met face-to-face and the other members were consulted via email and telephone. Single parameter danger signs were chosen rather than a composite score due to simplicity and to enable specific treatment directives.

### Outcome measures

The primary endpoint of the study was acute treatment, defined as the stipulated treatment in the VSDT protocol at the same time, or in the hour following, documentation of a danger sign. Acute treatments were assessed using two methods: Acute treatments at admission and acute treatments in ICU. Acute treatments at admission were following danger signs noted at arrival to the ICU. Acute treatments in ICU were following danger signs at any point during care in the ICU.

The aim was to study all the danger signs and acute treatments in the VSDT protocol (Fig 1). However, the following were excluded prior to the study: 1) if a danger sign for low conscious level was documented and the patient had an endo-tracheal tube in-situ, the danger sign was discounted as no action is stipulated when the airway is protected; 2) If a danger sign for abnormal respiratory rate or low oxygen saturation was documented and the patient was receiving 100% oxygen, the danger sign was discounted as the stipulation of increasing oxygen was not possible; 3) the danger sign for a high level of inspired oxygen had no corresponding directive and was omitted. Moreover, once data collection began it became clear that two further adjustments were required 1) airway sounds were not sufficiently documented so the danger sign was omitted from analyses; 2) patient position was not sufficiently documented so positional changes for a respiratory or circulatory danger sign could not be assessed.

### Secondary endpoints

All the patients were followed up until discharge or death to assess length of stay in the ICU and in-hospital mortality.

### Study population

The study took place between 11th November 2012 and 31st May 2015. For acute treatments at admission, data from all patients over 16 years who were admitted and had completed their care in the ICU during these dates were included. Patients who had part of their care during the implementation period of 10th March and 4th April 2014 and those missing admission vital signs or hospital outcome were excluded.

For analysis of acute treatments in ICU a sample of danger signs from the ICU observation charts was required. In a pilot study in the ICU in 2012, 18% of vital sign observations were danger signs, of which 6% were followed by an acute treatment [30]. For an adjudged clinically relevant quadrupling in acute treatments, 72 danger signs from 318 vital signs observations were needed in each group. We extracted all danger signs and treatments from three months pre-implementation (May 2013, September 2013, January 2014) and three months post-implementation (May 2014, September 2014, January 2015), as a compromise between power, logistical feasibility given time constraints, and a spread of observations throughout the study.

### Data acquisition

Danger signs and acute treatments were extracted from the charts. A detailed description of data extraction can be seen in our previous study [29]. The time-of-day when danger signs were detected were noted to determine practices in the mornings (07:00–14:59), afternoons (15:00–22:59) and nights (23:00–06:59).

### Implementation

The protocol was implemented between 10^th^ March and 4^th^ April 2014. Standard operating procedures requiring the use of the protocol were written and signed by the hospital administration. Information was spread to all departments and clinicians in the hospital. All nurses and doctors working in the ICU were trained in a one-day seminar followed by bedside teaching in the unit. Posters of the protocol were put up in the ICU. Two doctors and four nurses were designated as local facilitators and received extra training to be able to reinforce the use of the protocol during the post-implementation period. The protocol was attached to the patients’ observation charts daily during the post-implementation period. Fortnightly communication between the research team in Sweden and Tanzania facilitated logistics and allowed feedback on performance and outcomes. Short supervisory visits by members of the Swedish team were conducted: five in the pre-implementation and four in the post-implementation periods.

### Statistical analysis

Stata (Release 12, StataCorp, Texas) was used. Data were summarized with mean, median, standard deviation and inter-quartile ranges for numerical variables, and frequency tables for categorical variables. Pre and post-implementation groups were compared using chi^2^ for categorical and t-test or Wilcoxon rank sum test for numerical variables. Prevalence ratios (PR) comparing outcomes were used together with odds ratios (OR) as they are more intuitively interpretable [31, 32]. PRs were calculated using generalised linear models, with estimates calculated by generalised estimating equations when data were clustered within patients. ORs were estimated using logistic regression and mixed-effects models when data were clustered within patients. Multivariable models were built to adjust for age and made only minimal changes to results, and so crude ratios are not shown. Other patient factors (admission ward, planned/unplanned admission, respiratory support, sex) were not included as they were not considered to be confounders due to an absence of association with the implementation–i.e. they were equally distributed in the pre and post-implementation groups (Table 1). A variable of time between study-start and admission was used for each patient to analyse the effect of underlying time trends, as used by Howell and colleagues [33]. We focused mortality analyses on the circulation danger signs as we considered a-priori that respiratory treatments were already used in the ICU at Muhimbili due to the frequent use of mechanical ventilation. Differences in the secondary endpoints were subjected to further analyses to look for the effects of confounding and effect modification. Odds ratios and prevalence ratios were given with 95% confidence intervals and p-values <0.05 were considered statistically significant.

**Table 1:** Patient Characteristics. Age, sex, admitting ward, admitting specialty, illness severity and length of stay for all patients over 16 years in the ICU.

			Before	After	
			n = 269	n = 178	
			n (%)	n (%)	p-value
**Age (median (IQR) in years)**			35 (24–50)	40 (29–53)	0.01^1^
**Female**			146 (54%)	89 (50%)	0.38^2^
		Ward in Muhimbili Hospital	53 (20%)	38 (21%)	
**Admitted to ICU from**		Emergency Room	48 (18%)	30 (17%)	0.92^2^
		Operating Theatre	160 (59%)	105 (59%)	
		Other hospital	8 (3%)	5 (3%)	
		**Any planned admission**	**69 (26%)**	**39 (22%)**	0.37^2^
**Planned**	**Post-op**	ENT	37 (14%)	24 (13%)	-
**admissions**	**elective**	Thyroid	16 (6%)	13 (7%)	-
	**surgery**	Abdominal/gynaecological	7 (3%)	1 (1%)	-
		Thoracic	9 (3%)	1 (1%)	-
	**Any unplanned admission**		**200 (74%)**	**139 (78%)**	-
		**Any emergency surgery**	**113 (42%)**	**89 (49%)**	0.14^2^
		Abdominal	68 (25%)	59 (33%)	-
		ENT	5 (2%)	1 (1%)	-
	**Post-op**	Obstetrics/Gynaecology	30 (11%)	26 (14%)	-
	**emergency**	Thyroid	2 (0.8%)	0 (0%)	-
	**surgery**	Breast	1 (0.4%)	0 (0%)	-
**Unplanned**		Urological/Renal	2 (0.8%)	1 (1%)	-
**admissions**		Thoracic	5 (2%)	2 (1%)	-
		**All medical** ^a^	**87 (32%)**	**50 (28%)**	0.34^2^
		Obstetrics/Gynaecology	12 (4.5%)	4 (2%)	-
		Internal Medicine	52 (19%)	29 (17%)	-
	**Medical** ^a^	Infectious Diseases	17 (6%)	12 (7%)	-
		Foreign body / poisoning	5 (2%)	5 (3%)	-
		Unknown	1 (0.5%)	0 (0%)	-
		Receiving Oxygen^b^	221/250(88%)	133/160 (83%)	0.13^2^
		Intubated	202 (75%)	141 (79%)	0.31^2^
		Mechanical ventilation	137 (51%)	82 (46%)	0.31^2^
**Illness**		Any Danger Sign	186 (69%)	117 (66%)	0.4^2^
**Severity**		Number of Danger Signs (Median(IQR))	1 (0–2)	1 (0–2)	0.69^1^
		Heart Rate Danger Sign	37 (13.8%)	17 (9.6%)	0.18^2^
		Systolic Blood Pressure Danger Sign	39 (14.5%)	26 (14.6%)	0.98^2^
		Patients with NEWS ≥ 7	168 (62%)	108 (60.7%)	0.7^2^
		NEWS (Mean (SD))	7.8 (3.0)	7.5 (2.9)	0.38^3^
**Length of stay in ICU in days (Median(IQR))**			1.8 (0.9–6.4)	2.9 (1.1–8.0)	0.004^1^

IQR interquartile range

ICU intensive care unit

ENT ear, nose & throat

IQR interquartile range

NEWS national early warning score

SD standard deviation

^1^ wilcoxon rank-sum

^2^ chi^2^

^3^ t-test

^a^ medical = not post-operative

^b^ data missing on oxygen therapy at admission for 29 patients and 27 patients in pre and post groups respectively.

### Ethical considerations

Ethical clearance was granted by the National Institute for Medical Research in Tanzania (NIMR/HQ/R.8a/Vol.IX/1606), Muhimbili University of Health & Allied Sciences (MU/DRP/AEC/Vol.XVI/125) and the Ethical Review Board in Stockholm (EPN/2015/673-31/2). Permission for the study was granted by The Tanzanian Commission for Science and Technology and by MNH. As the study was part of quality improvement in the ICU and the data was anonymised for analysis, individual patient consent was waived. The European Critical Care Research Network of the European Society of Intensive Care Medicine endorsed the study

## Results

### General characteristics

**Fig 2:**
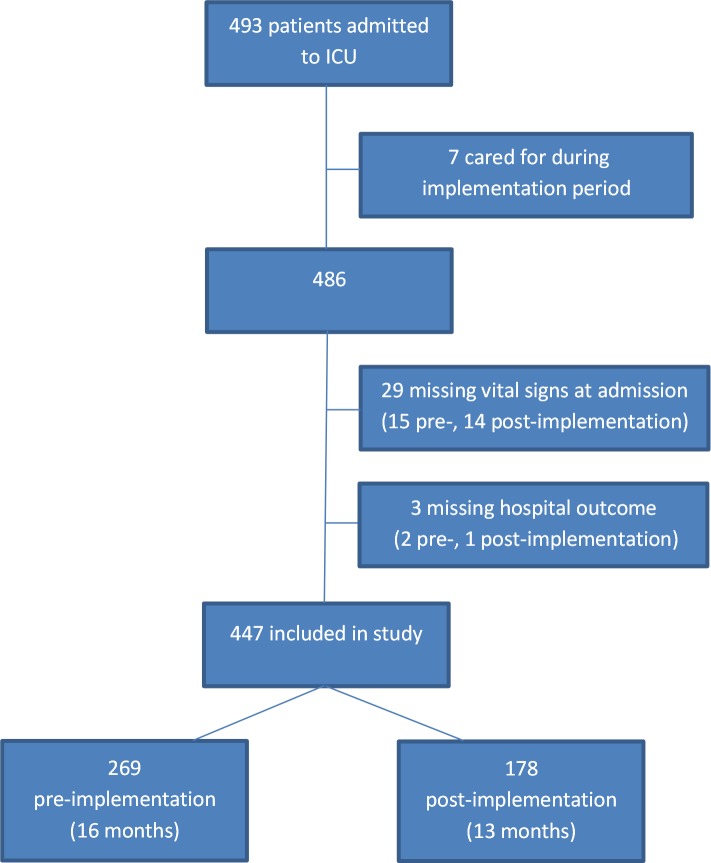
Study flow chart.

493 patients were admitted to the ICU during the study period. After excluding patients missing admission vital signs or outcomes, 447 patients were included in the study: 269 pre-implementation and 178 post-implementation (Fig 2). The post-implementation patients were older than the pre-implementation patients, but other baseline characteristics and illness severity were similar (Table 1). At admission, 69% of pre-implementation and 66% of post-implementation patients had one or more danger sign (p = 0.40). Post-implementation, the median length of stay was longer (2.9 days compared to 1.8 days, p = 0.004).

The six-month sample used for acute treatments in ICU contained 2603 danger signs: 1304 danger signs pre-implementation, extracted from 58 patients’ observation charts, and 1299 danger signs from 59 patients post-implementation. Abnormal respiratory rate (38%), heart rate (37%) and systolic blood pressure (20%) were the most common danger signs. Abnormal conscious level (3%) and oxygen saturation (2%) were uncommon.

### Acute treatments

Acute treatment of all danger signs at admission was higher post-implementation (62 of 85 danger signs, 72.9%) than pre-implementation (36 of 156 danger signs, 23.1%, PR 3.2 (2.2–4.5)) (Table 2). Acute treatment of each danger sign at admission was significantly higher post-implementation than pre-implementation. The largest increase was for hypotension: 80.0% (21/26) of patients received an acute treatment of intravenous fluid post-implementation, compared to 36.1% (13/36) pre-implementation (PR 2.2 (1.4–3.5)).

**Table 2:** Acute treatments. Acute treatments carried out when danger signs detected at admission to ICU and during care in ICU pre and post implementation.

		Acute treatments					Adjusted^1^		Adjusted^1^	
		pre-		post-			Odds		Prevalence	
		implementation		implementation			Ratio		Ratio	
		n/n_tot_	%	n/n_tot_	%	p-value	(95% CI)		(95% CI)	
	**All Danger Signs**	**36/156**	**23.1**	**62/85**	**72.9**	**<0.001**	**9.2** ^2^	**4.9–16.5**	**3.2** ^3^	**2.2–4.5**
	GCS<9	4/22	18.2	8/11	72.7	0.002	10.6^4^	1.8–61.5	4.0^5^	1.5–10.4
**At**	Respiratory Rate <8 or >30 / min	3/47	6.4	8/17	47.1	<0.001	14.8^4^	3.1–70.9	7.4^5^	2.2–24.6
**admission**	Oxygen Saturation <90%	9/23	39.1	12/14	85.7	0.006	10.5^4^	1.8–62.2	2.2^5^	1.3–3.8
	Heart Rate <40 or >130 beats/min	7/28	25.0	13/17	76.5	0.001	9.9^4^	2.4–41.1	3.1^5^	1.5–6.1
	Systolic Blood Pressure <90 mmHg	13/36	36.1	21/26	80.0	<0.001	7.6^4^	2.3–25.4	2.2^5^	1.4–3.6
	**All Danger Signs**	**38/1304**	**2.9**	**216/1299**	**16.6**	**<0.001**	**7.9** ^2^	**4.1–15.2**	**4.9** ^3^	**2.9–8.3**
	GCS<9	3/78	3.9	0/0	-	-	-	-	-	-
**During**	Respiratory Rate <8 or >30 / min	4/561	0.7	11/419	2.6	0.016	4.0^2^	1.2–12.8	4.1^3^	1.3–12.6
**care in**	Oxygen Saturation <90%	0/17	0.0	4/25	16.0	0.083	-	-	-	-
**ICU**	Heart Rate <40 or >130 beats/ min	22/426	5.2	94/549	17.1	<0.001	4.2^2^	1.7–10.0	2.7^3^	1.5–4.8
	Systolic Blood Pressure <90 mmHg	9/222	4.1	107/306	35.0	<0.001	14.5^2^	5.0–42.4	6.4^3^	2.5–16.2

n/n_tot_ number of times protocol adhered to divided by number of danger signs

ICU Intensive Care Unit

GCS Glasgow Coma Score

^1^ odds ratios and prevalence ratios adjusted for age

^2^ odds ratios calculated with mixed-effects models when data were clustered within patients

^3^ prevalence ratios calculated with generalised estimating equations when data were clustered within patients

^4^ odds ratios calculated with logistic regression

^5^ prevalence ratios calculated with generalised linear models.

**Fig 3:**
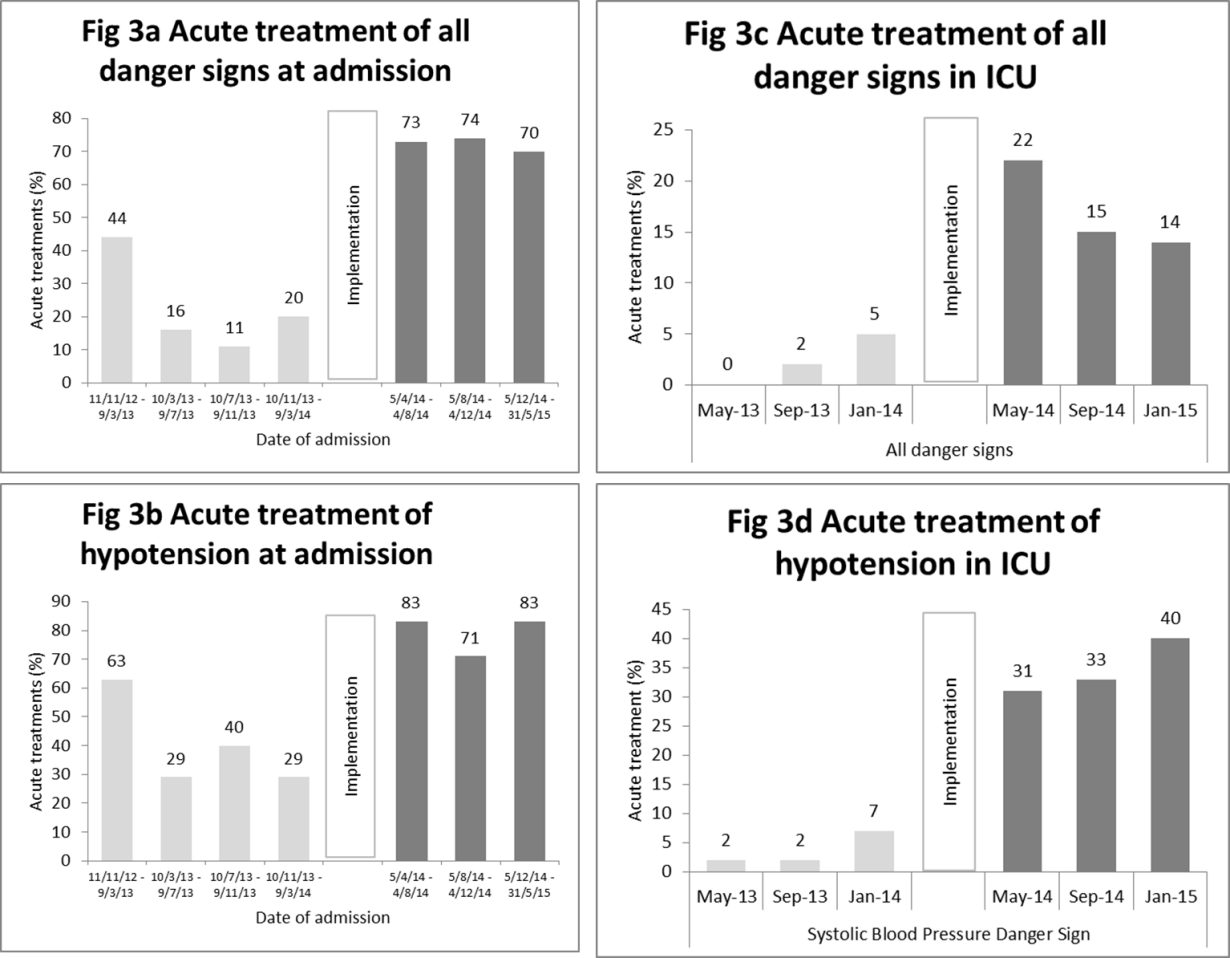
Acute treatments in the ICU over time.

Acute treatment for all danger signs in ICU was higher post-implementation (216 of 1299, 16.6%) than pre-implementation (38 of 1304, 2.9%) (PR 4.9 (2.9–8.3)). Again, the largest increase was for hypotension: 35.0% (107/306) of patients received fluids post-implementation, compared to 4.1% (9/222) pre-implementation (PR 6.4 (2.5–16.2)). The increase in acute treatments both at admission and in ICU occurred at the time of the implementation (Fig 3).

### Time-of-day and underlying time trend

There were no statistically significant differences in the proportions of patients admitted during the morning (49.4% pre and 51.7% post implementation), afternoon (36.4% pre and 37.6% post implementation), and night (14.1% pre and 10.7% post implementation, p = 0.56). Acute treatments *at admission* did not vary significantly by time-of-day pre or post implementation. Acute treatments *in ICU* did not vary significantly by time-of-day pre-implementation, but post-implementation, acute treatments in ICU were significantly higher in the morning (95 of 415, 22.9%) compared to the afternoon (69 of 447, 15.4%, PR 1.10 (1.02–1.18)) and night (52 of 437, 11.9%, PR 1.14 (1.07–1.22)). A variable for time between study-start and patient admission did not have a significant association with acute treatment at admission in the multivariable analyses and was not included in the presented models.

### Mortality

Overall in-hospital mortality rates were unchanged (49.8% pre-implementation, 49.4% post-implementation, p = 0.94) (Table 3). In patients admitted with hypotension, in-hospital mortality was lower post-implementation (69.2%) compared to pre-implementation (92.3%, p = 0.02 PR 1.3 (1.02–1.7)). There were significantly more hypotensive patients admitted after emergency surgery in the post-implementation group as compared to pre-implementation, but emergency surgery was not independently associated with mortality, and analysis did not reveal any effect modification. The pre and post-implementation hypotensive patients were similar for all other variables (data not shown). To prevent the death of one patient admitted with hypotension, the number-needed-to-treat with VSDT was 4.3.

**Table 3:** In-hospital mortality. In-hospital mortality in pre and post-implementation patients, by the presence of danger signs.

		In-hospital mortality					Adjusted		Adjusted	
		pre-		post-			Odds		Prevalence	
		implementation		implementation			Ratio^2^		Ratio^3^	
		n/n_tot_	%	n/n_tot_	%	p-value^1^	(95% CI)		(95% CI)	
	**All**	**135/269**	**49.8**	**88/178**	**49.4**	**0.94**	**0.9**	**0.6–1.4**	**0.9**	**0.8–1.1**
	One or more Danger Sign at admission	119/187	63.6	72/117	61.5	0.71	0.9	0.5–1.4	0.9	0.8–1.1
**All**	Heart rate Danger Sign at admission	30/37	81.1	12/17	70.6	0.39	0.6	0.1–2.1	0.8	0.6–1.2
**patients**	Systolic Blood Pressure Danger Sign at admission	36/39	92.3	18/26	69.2	0.02	0.2	0.04–0.8	0.75	0.6–0.98
	Heart rate Danger sign at any time in ICU^4^	8/10	80.0	7/10	70.0	0.48	2.1	0.3–15.4	0.8	0.5–1.4
	Systolic Blood Pressure Danger sign at any time in ICU^4^	10/11	90.9	13/16	81.3	0.49	0.4	0.03–5.2	0.9	0.7–1.2
** Unplanned admissions**		**129/200**	**64.5**	**84/139**	**60.4**	**0.45**	**0.5**	**0.07–3.5**	**0.9**	**0.8–1.1**

n/n_tot_ number of deaths divided by number of patients

^1^ chi2

^2^ odds ratios calculated by logistic regression, adjusted for age

^3^ prevalence ratios calculated by generalised linear models adjusted for age

^4^ in the sample of 6 months of patient charts.

### Other changes to ICU

The interviews with the head nurse in ICU did not reveal any major changes to the personnel, staffing rota, equipment, consumables or drug availability in the ICU during the study period. To her knowledge, admission routines and hospital practices and resources for the management of sick patients were not changed.

## Discussion

We have found that the introduction of the VSDT protocol improved the acute care of patients with abnormal vital signs in an ICU in a LIC. The multi-faceted implementation method resulted in an increase in acute treatment of danger signs at admission and during care in the ICU by three times and five times respectively. Mortality rates were reduced in patients admitted to the ICU with hypotension from 92.3% to 69.2%. In-hospital mortality for the whole patient group was unchanged.

The VSDT protocol standardises emergency care for patients with abnormal physiological parameters. It facilitates the identification of at-risk patients and stipulates the treatments when derangements are detected (goal-directed therapy). Identifying at-risk patients using vital signs has been implemented in many parts of the world. In the United Kingdom the National Early Warning Score is used throughout the country [23]. In Karolinska hospital in Sweden, single parameter criteria are used [27], and in Tanzania vital sign abnormalities were found to be associated with mortality [28]. Goal-directed therapies have become a mainstay in critical care since a landmark study from USA in 2001 reduced mortality from sepsis by 30% [34, 35]. The International Surviving Sepsis Campaign has standardised care for critically ill patients with sepsis, leading to a mortality reduction from 57% to 38% in one study [36, 37]. While some studies have not found survival benefits of goal-directed strategies, this is likely due to improved early detection and treatment in the control arms as a result of knowledge gleaned from earlier goal-directed studies [25, 38, 39].

International guidelines are not always possible to implement in LICs, necessitating the development of context-appropriate recommendations [11, 40, 41]. The sparse data on protocols for critical care in LIC shows conflicting results. In Uganda, improving early monitoring and management of sepsis led to a reduction in 30-day mortality [42]. A simplified protocol for severe sepsis was trialled in Zambia and showed no mortality benefit, however the study population included a large proportion of patients suffering from the sub-acute or chronic infections tuberculosis or HIV [26]. The VSDT protocol is simple and pragmatic and was designed for all adult patients, irrespective of underlying diagnosis. An underlying principle of the VSDT protocol is task-shifting of care from physicians to nurses enabling quick care where access to physicians is limited. Task-shifting has been effective and safe in emergency obstetric care [43], HIV-treatment [44] and surgical care [45]. The VSDT protocol could be adjusted or overruled by the treating clinician and was not designed to replace medical judgement or management.

There is some evidence that fluid boluses to critically ill patients may be harmful. In African children with febrile illness and signs of poor perfusion 20–40ml/kg fluid boluses were associated with increased mortality [46]. In Zambia the sepsis protocol trial including 2000ml fluid boluses was stopped early as patients with hypoxaemic respiratory distress might have been at increased risk from the intervention [26]. However, it remains standard practice in critical care to treat shock with intravenous fluid [35, 36, 41] and the VSDT protocol uses smaller volume boluses to minimise risks. The reportedly pervasive practice of minimal resuscitation [47] and our previous findings that over two-thirds of patients in the ICU at Muhimbili had danger signs and that few received acute treatments [48, 49] was our justification for a simple protocol.

The implementation method was both multifaceted, as recommended by others [50], and pragmatic to enable transferability to similar settings. Researchers from Sweden did not spend long periods of time in Tanzania: the focus was on local facilitators and local ownership. Costs were low: no new personnel were employed, no new equipment or medicines were introduced and the training was conducted by staff working pro bono. We were unable to collect data on intravenous fluid utilisation but an increase post-implementation was likely. A bag of intravenous fluids costs the hospital only $0.30, so extra costs were unlikely to be substantial.

Although several-fold improvements were seen post-implementation, acute treatments following all danger signs in the ICU were only 17%, and fluids were administered in only one-third of hypotensive episodes. There could be several reasons for this somewhat disappointing adherence to the protocol: the protocol’s simplification of complex medical situations, a failure of implementation so staff did not fully understand the use of the protocol, a lack of buy-in by decision makers and influential doctors or more generally a low organisational capacity for change [51, 52]. A 25% reduction of in-hospital mortality for patients admitted with hypotension is a promising finding. Although rates of acute treatments at admission were similar in the day, afternoon and night shifts, the acute treatments in ICU were markedly lower in the afternoons and nights post implementation. The low adherence in ICU, particularly in the afternoons and nights, may explain the unchanged overall mortality rates. Length of stay in the ICU increased post-implementation, which may indicate that the intervention had some effect at prolonging survival.

A limitation of any before-and-after study is the possibility of temporal confounding: we cannot be certain that the effects are due to the implementation of the protocol–there could be other factors that differ between the pre and post implementation periods. There may have been differences in the patients’ diagnoses, which we attempted to account for with data on admitting wards and specialties. However, we were not able to collect reliable data on final diagnoses or co-morbidities. We did find an age difference–the post-implementation patients had a median age five years older than the pre-implementation patients. This could explain the lack of a reduction in mortality for the whole cohort–the older patients were likely to have worse outcomes than the younger patients, which we had seen in a previous study [29]. There could also have been differences in the care given on the unit, independent of our intervention. However, the monthly interviews with the head nurse in ICU did not reveal other changes to the ICU, and moreover the improvements in acute treatments coincided with the implementation period, as can be seen in Fig 3. One further limitation was the use of in-hospital mortality as an endpoint–this may have misclassified terminally ill patients who were discharged home to die.

Further research should assess the VSDT protocol in other ICUs and wards in Tanzania and other LICs and the cost-effectiveness of critical care in LICs [1, 53]. Qualitative research is required to explore the facilitators and barriers to quality critical care and the optimal ways to implement improved clinical practices for the identification and treatment of the critically ill.

## Conclusion

The introduction of a vital signs directed therapy protocol improved the acute treatment of abnormal vital signs in an ICU in a LIC. Mortality rates were reduced for patients with hypotension at admission but not for all patients.
